# Reply to Zrelovs et al. PVJ1 Is Not the First Tailed Temperate Phage Infecting Bacteria from Genus *Psychrobacillus*. Comment on “Liu et al. Isolation and Characterization of the First Temperate Virus Infecting *Psychrobacillus* from Marine Sediments. *Viruses* 2022, *14*, 108”

**DOI:** 10.3390/v14050866

**Published:** 2022-04-22

**Authors:** Wang Liu, Xiaowei Zheng, Xin Dai, Zhenfeng Zhang, Wenyan Zhang, Tian Xiao, Li Huang

**Affiliations:** 1 State Key Laboratory of Microbial Resources, Institute of Microbiology, Chinese Academy of Sciences, Beijing 100101, China; liuwang17@mails.ucas.ac.cn (W.L.); zhengxw@im.ac.cn (X.Z.); daix1970@163.com (X.D.); zhangzf@im.ac.cn (Z.Z.); 2 College of Life Science, University of Chinese Academy of Sciences, Beijing 100049, China; 3 Key Laboratory for Marine Ecology and Environmental Sciences, Institute of Oceanology, Chinese Academy of Sciences, Qingdao 266071, China; zhangwy@qdio.ac.cn (W.Z.); txiao@qdio.ac.cn (T.X.); 4 Center for Ocean Mega-Science, Chinese Academy of Sciences, Qingdao 266071, China; 5 Laboratory for Marine Ecology and Environmental Science, Qingdao National Laboratory for Marine Science and Technology, Qingdao 266273, China

We thank Zrelovs et al. [1] for drawing our attention to the fact that they deposited the first virus genomes (i.e., MT325768.1 and MT410774.1) in the GenBank database, which they indicated in the database entries were from *Psychrobacillus* phages, termed Perkons and Spoks, respectively, ahead of our submission of the PVJ1 genome sequence. They speculated correctly that we did not perform a sequence database search with *Psychrobacillus* phage-related queries immediately before the submission of our paper [2]. The lack of significant genome sequence similarity also prevented us from identifying Perkons and Spoks during our frequent searches with the PVJ1 sequence. We argue, however, that there is a significant flaw in their claim that they reported the first *Psychrobacillus* phages for a simple reason that the claim was not substantiated by peer-reviewed evidence. No experimental data were published in peer-reviewed literature to relate their deposited genomes to *Psychrobacillus*. To validate their claim, the authors should have demonstrated in peer-reviewed literature that the two genomes were isolated from virions (Perkons and Spoks) released from or capable of infecting purified *Psychrobacillus* strains or identified from the genomes of *Psychrobacillus* strains. Therefore, in our view, their claim was unproven.

The authors are apparently aware of the flaw with that claim as they explained in their comment: ‘Despite the fact that there is a large fraction of complete annotated phage genome entries publicly available that still do not have an article or at least a genome announcement from the peer-reviewed literature linked to them for a variety of reasons (including *Psychrobacillus* phages Perkons and Spoks), we believe it is incorrect to ignore the existence of such entries when analyzing the place of any newly isolated phage within the context of known phages, regardless of the phages not being mentioned anywhere in the peer-reviewed literature’ [1]. Furthermore, they tried to fill the gaps in the data by providing some experimental details relevant to the isolation of Perkons and Spoks in their comment [1]. Obviously, this latest explanatory information, though still incomplete, should have been presented in a peer-reviewed fashion when they claimed to have found the first *Psychrobacillus* phages. Somewhat surprisingly, they made a new unsubstantiated claim, which did not exist in their original GenBank entries, in their comment. Since our paper indicates that PVJ1 is the first temperate virus isolated that infects *Psychrobacillus*, they argued in their comment that ‘PVJ1 still remains the third temperate tailed virus… following the temperate *Psychrobacillus* siphophages Perkons and Spoks’. However, no phage induction experiments were mentioned, and no evidence of the integration of either Spoks or Perkons DNA into the *Psychrobacillus* genome was presented in the Supplementary Materials attached to their comment (https://www.mdpi.com/1999-4915/14/3/495/s1?version=1646035139). Therefore, how can one accept the claim that Perkons and Spoks are temperate *Psychrobacillus* phages? 

All things considered, we would like to clarify the following points in response to their comment. Zrelovs et al. deposited the first virus genomes claimed to be from *Psychrobacillus* phages, but the claim remains to be proved with peer-reviewed evidence. Additionally, our paper represents the first peer-reviewed publication on the isolation and characterization of a *Psychrobacillus* phage.
